# Does a SLAP lesion affect shoulder muscle recruitment as measured by EMG activity during a rugby tackle?

**DOI:** 10.1186/1749-799X-5-12

**Published:** 2010-02-25

**Authors:** Ian G Horsley, Lee C Herrington, Christer Rolf

**Affiliations:** 1 Sheffield Centre for Sports Medicine, University of Sheffield, UK; 2 Centre of Rehabilitation and Human Performance Research, University of Salford, UK

## Abstract

**Background:**

The study objective was to assess the influence of a SLAP lesion on onset of EMG activity in shoulder muscles during a front on rugby football tackle within professional rugby players.

**Methods:**

Mixed cross-sectional study evaluating between and within group differences in EMG onset times. Testing was carried out within the physiotherapy department of a university sports medicine clinic. The test group consisted of 7 players with clinically diagnosed SLAP lesions, later verified on arthroscopy. The reference group consisted of 15 uninjured and full time professional rugby players from within the same playing squad. Controlled tackles were performed against a tackle dummy. Onset of EMG activity was assessed from surface EMG of Pectorialis Major, Biceps Brachii, Latissimus Dorsi, Serratus Anterior and Infraspinatus muscles relative to time of impact. Analysis of differences in activation timing between muscles and limbs (injured versus non-injured side and non injured side versus matched reference group).

**Results:**

Serratus Anterior was activated prior to all other muscles in all (P = 0.001-0.03) subjects. In the SLAP injured shoulder Biceps was activated later than in the non-injured side. Onset times of all muscles of the non-injured shoulder in the injured player were consistently earlier compared with the reference group. Whereas, within the injured shoulder, all muscle activation timings were later than in the reference group.

**Conclusions:**

This study shows that in shoulders with a SLAP lesion there is a trend towards delay in activation time of Biceps and other muscles with the exception of an associated earlier onset of activation of Serratus anterior, possibly due to a coping strategy to protect glenohumeral stability and thoraco-scapular stability. This trend was not statistically significant in all cases

## Background

Several authors have highlighted that shoulder injuries are becoming more severe within professional rugby [1-3]. Tackling or being tackled is responsible for a majority of these reported shoulder injuries [4,5,3]. For practitioners of sports medicine, rugby, both the rugby League and Union codes, appear to have a high risk of injury per exposure time [6-8]. This figure is around 100 injuries per 1000 hours or play, which is significantly greater than in soccer reporting 26 injuries per 1000 hours. The explanation for this high incidence is probably due to the high number of collisions during competition, resulting in musculoskeletal injury [9].

Sports injuries are a multi-risk phenomena [10] and the intricacy of the relations among them, mean that identifying underlying mechanisms poses a challenge to epidemiologists [11,12]. Potential risk factors to injury within sportsmen have been classified into intrinsic and extrinsic [13]. Intrinsic factors are specific to the individual, and include age, sex, anthropometric characteristics, fitness, psychological characteristics, health status, and injury history. These factors cannot be corrected quickly [6]. Extrinsic factors are environmental factors out of direct control of the sportsman [6] and include the nature of the sport, environmental conditions, and equipment. The identification of risk factors associated with the effect of the injury on subsequent participation may be as important in understanding how to reduce the burden of injuries on sports participants as identifying factors associated with the injury incidence rate [14].

The tackle appears to be the phase of play associated with the greatest risk of injury overall [3,15,16], yet there appears to be scant published research regarding the anatomical and biomechanical stresses that are placed on the shoulder and surrounding structures during its execution. Electromyography (EMG) has been utilized as a tool for analyzing the function of muscles since 1944 [17]. It has since been used to assess muscle function in both normal and injured subjects. Several authors have analyzed muscle recruitment activity around the lumbar spine and abdomen in patients with and without low back pain [18-20] cervical muscle function [21,22] knee and patello femoral joint [23-25] and there are a few studies related to the shoulder girdle [26-28] who all showed alterations in muscle recruitment patterns around the shoulder in subjects with instability.

In many sports, precise motor acquisition and rapid reaction time are important in preventing injury to the joint. An altered interaction between the dynamic and passive stabilizers may predispose a sportsman to an increased incidence of joint disruption [29]. Delay in the reaction time of the neuromuscular system is termed electromechanical delay (EMD). This is defined as the time delay between the onset of muscle activity and the onset of force generation [30]. If present this could allow for uncontrolled motion at a joint, resulting in damage to the passive structures of the joint during activity [31].

Lesions involving the superior labrum and the origin of the tendon of the long head of the Biceps Brachii muscle, the biceps anchor, can cause shoulder pain and instability. Andrews *et al*., (1985) [32] first described labral injuries in throwing athletes initially reporting tearing of the anterosuperior labrum from the glenoid, and in 1990, Snyder et al. [33] portrayed the superior labral anterior posterior (SLAP) lesion. It represents an injury to the superior labrum that begins posteriorly and extends anteriorly, and it often includes the origin of the biceps tendon.

The superior glenoid labrum and the long head of the Biceps contribute to the stability of the glenohumeral joint [32,34,35]. Previous electromyographic (EMG) studies have identified that due to this action of the long head of Biceps as a dynamic stabilizer of the glenohumeral joint SLAP, lesions can occur as a result of chronic overuse from forceful contraction of the Biceps tendon [36,37]. Strain has also been shown to increase within the superior labrum of cadavers as the tension is increased within the tendon of Biceps, as the humerus moves from adduction towards 90 degrees of abduction, as seen within rugby players as they carry out a tackle [38].

Several authors have evaluated reflex muscle activity in unstable shoulders. Myers et al., (2004) [39] utilizing a combination of surface electromyography (sEMG) and indwelling electrodes, compared the mean activation of glenohumeral joint muscles when testing reflex action in the apprehension position with a population of subjects demonstrating anterior glenohumeral instability, and their matched controls. They found suppressed rotator cuff co-activation, slower Biceps Brachii activation, and decreased Pectorialis Major and Biceps Brachii, and compared them to 12 similar athletes who did not display signs of instability. They demonstrated an imbalance within the shoulder muscles (Biceps, Supraspinatus, Infraspinatus, Pectorialis Major, Subscapularis, Latissimus Dorsi and Serratus Anterior) of the unstable shoulders during the throwing activity. There was a mild increase in activity of Pectorialis Major, Latissimus Dorsi and Serratus Anterior, especially at the extreme of external rotation in abduction. They suggested that during rehabilitation, emphasis should be placed on the scapular protractor muscles.

Superior labral lesions may also occur in an acute setting due to rapidly experienced eccentric loads of the biceps tendon, which produces traction to the tendon's attachment at the labrum [34]. Within a retrospective review of 700 arthroscopies, described by [33] Snyder et al., (1990), 27 patient who were found to have SLAP lesions, described a common mechanism of injury producing a compression force to the shoulder, most often as a result of a fall onto an outstretched arm, with the shoulder in the position of abduction and slight forward flexion at the time of impact. In this position, it is postulated, that the tendon of biceps becomes pinched between the humeral head and the glenoid resulting in a traumatic disruption of the superior labrum. Associated injuries include rotator cuff tears [40,41], chondral lesions [42,43], and instability of the glenohumeral joint [44,35].

Pagnani *et al*. [35] found that simulated type II SLAP lesions result in increased glenohumeral translations in both the anteroposterior and superoinferior directions, and in addition,[44] Burkart *et al*. demonstrated in a cadaveric study, that the torsional rigidity of the shoulder was diminished after simulation of a type II SLAP lesion, and strain in the inferior glenohumeral ligament increased. Changes within the muscle activation pattern may predispose a player to, or be a consequence of, SLAP lesions. If so, rehabilitation programmes for the shoulders of professional rugby players may need to be altered.

This study aims to identify the muscle activation patterns within the shoulders of rugby players who have SLAP lesions, and compare them with the muscle activation patterns of their non injured shoulder and the muscle activation patterns within the shoulders of the control group

## Methods

Following Ethical approval by the University of Sheffield, 15 male full time, asymptomatic, professional rugby union players (mean age 22 +/- 1.4 years range 19-35) were recruited after giving written informed consent, along with 7 subjects who were clinically diagnosed with SLAP lesions.

Prior to the study, as part of their routine pre-season, screening programme, participants were evaluated by an orthopaedic consultant who specialized in shoulder trauma. Bilateral evaluation of all active, passive and resisted movements of the shoulder was a pre-requisite to the physical assessment. A battery of routine shoulder tests were incorporated into the examination in all subjects; these were O'Brien's test, Jobe's test, Hawkins-Kennedy test, Palm-up test, Compression rotation test, Apprehension-relocation test, across-body test, Gerber's lift-off test and Sulcus sign.

Results from the testing indicated the presence of a SLAP tear in 7 subjects, which were later confirmed during arthroscopy as all being grade II lesions. Inclusion criteria were; male, full time professional rugby players of at least two years duration, still participating fully in match day activities not experiencing pain when tackling, without a history of cervical, thoracic or lumbar spine, or lower limb injury within the last 12 months, and no previous surgical intervention to the presenting shoulder, and no complaints of contra lateral shoulder pain.

The electrodes were placed at specific sites where the muscle was superficial and the electrodes were placed parallel to the muscle fibers, preferably in the mid-line of the muscle belly between the nearest innervation zone and the musculotendinous junction, whereby the greatest signal amplitude can be detected.

The selected muscles were the ones which allowed for easy access for sEMG, and which have been reported to be responsible for global stabilization (Serratus Anterior, Infraspinatus and Biceps) and global mobilization (Pectorialis Major and Latissimus Dorsi) of the shoulder complex. Although the upper fibers of Trapezius were accessible, it was decided not to evaluate its activity, as it is also recruited in maintaining the cervical spine position and the alteration in head and neck position would have a cross talk effect on the sEMG activity which was recorded at the shoulder during the tackle.

### Serratus Anterior

**Figure 1:**
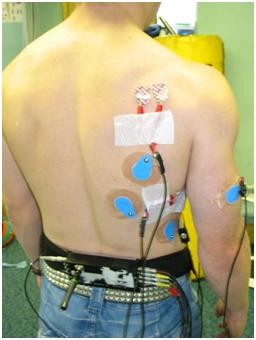
**Electrode Placement**.

(see figure 1) Two active electrodes were placed 2 cm apart, horizontally, just below the axillary area, at the level of the inferior angle of the scapula, just medial to the Latissimus Dorsi. Correct electrode placement was carried out by noting EMG activity during resisted protraction of the arm at 90 degrees flexion.

### Infraspinatus

**Figure 2:**
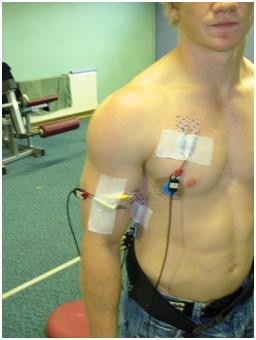
**Position for EMG Recording**.

(see figure 2). Following identification of the spine of the scapula, two electrodes were placed 2 cm apart parallel to and approximately 4 cm below the scapular spine on the lateral aspect of the infraspinous fossa. Correct electrode placement was carried out by noting the EMG activity during resisted lateral rotation of the arm whilst at 90 degrees abduction and with 90 degrees elbow flexion.

### Pectoralis Major

(Clavicular fibers) (see figure 1). Two active electrodes were placed 2 cm below the clavicle and medial to the axillary fold at an oblique angle 2 cm apart. Correct electrode placement was confirmed by noting the EMG signal during resisted humeral adduction at 90 degrees of forward flexion.

### Latissimus Dorsi

(see figure 2) Two active electrodes were placed 2 cm apart, approximately 4 cm distal to the inferior angle of the scapula, at an oblique angle of approximately 25 degrees. Correct electrode placement was confirmed by noting EMG signal activity during resisted humeral extension from 120 degrees forward flexion.

### Biceps Brachii

(see figure 1) Two active electrodes were placed 2 cm apart parallel to the muscle fibers in the centre of the biceps belly. Correct electrode placement was confirmed by noting the EMG signal during resisted elbow flexion

### Electromyography

Simultaneous recordings of the sEMG activity from the Pectorialis Major, Biceps Brachii, Latissimus Dorsi, Serratus Anterior and Infraspinatus muscles were made during the procedures outlined below. Prior to mounting the recording electrodes, the skin surface was prepared by light abrasion (Nuprep, SLE Ltd) and cleaning with alcohol swabs. Two silver/silver chloride bipolar electrodes (Medicotest UK, type N10A), with a 20 mm inter-electrode distance (centre to centre) were placed midline on one of the prepared muscle site locations outlined below. A ground electrode (Medicotest, UK, type Q10A), was placed at an electrically neutral site; the sternum. The sEMG was high and low pass filtered between 10 and 500 Hz respectively (Neurolog filters NL 144 and NL 134, Digitimer, UK), preamplified (×1000), (Neurolog remote AC preamplifier NL 824, Digitimer, UK), amplified (×2) (Neurolog isolation amplifier, NL 820, Digitimer, UK) and A/D converted at a rate of 2000 Hz (KPCI 3101, Keithley instruments, UK). To determine the sEMG signal on/off, a computer aided algorithm was used (Testpoint, Keithley instruments, UK) to allow a threshold value to be calculated from 3 standard deviations above baseline [45]. To ensure the validity of the computer derived sEMG onsets each trace was also visually inspected in order to ensure that movement artifact or other interference was not incorrectly identified as a muscle onset [45]. The impact of the tackle was determined from a pressure change detected in a pressure switch placed on the anterior superior aspect of the shoulder (marked **x **on Figure 1) and visual inspection of the EMG traces. The assessor of the sEMG data was blinded to which subjects had the proposed SLAP tears.

### Procedure

**Figure 3:**
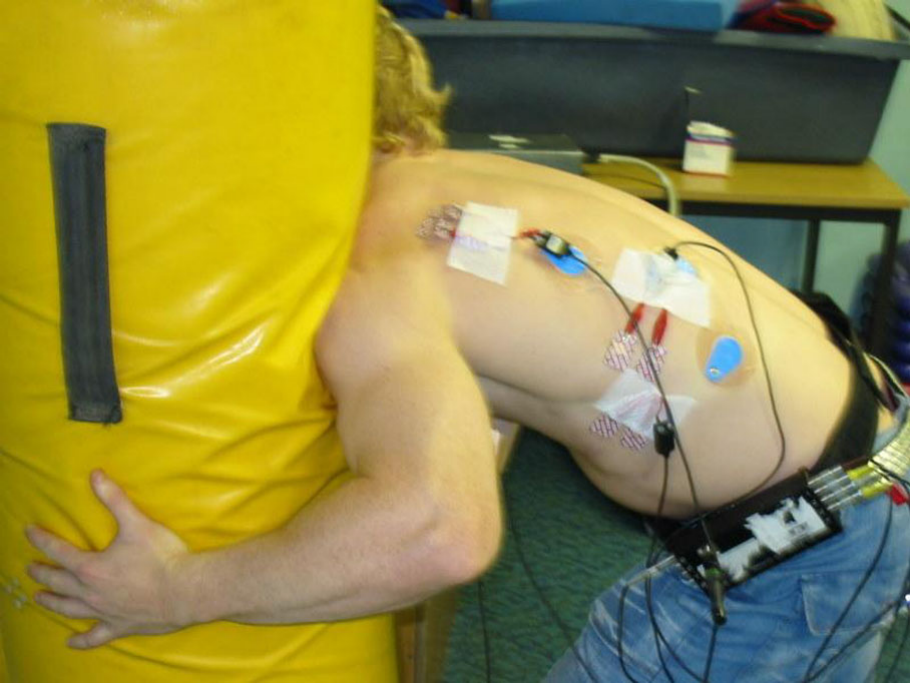
**Foot position at contact**.

**Figure 4:**
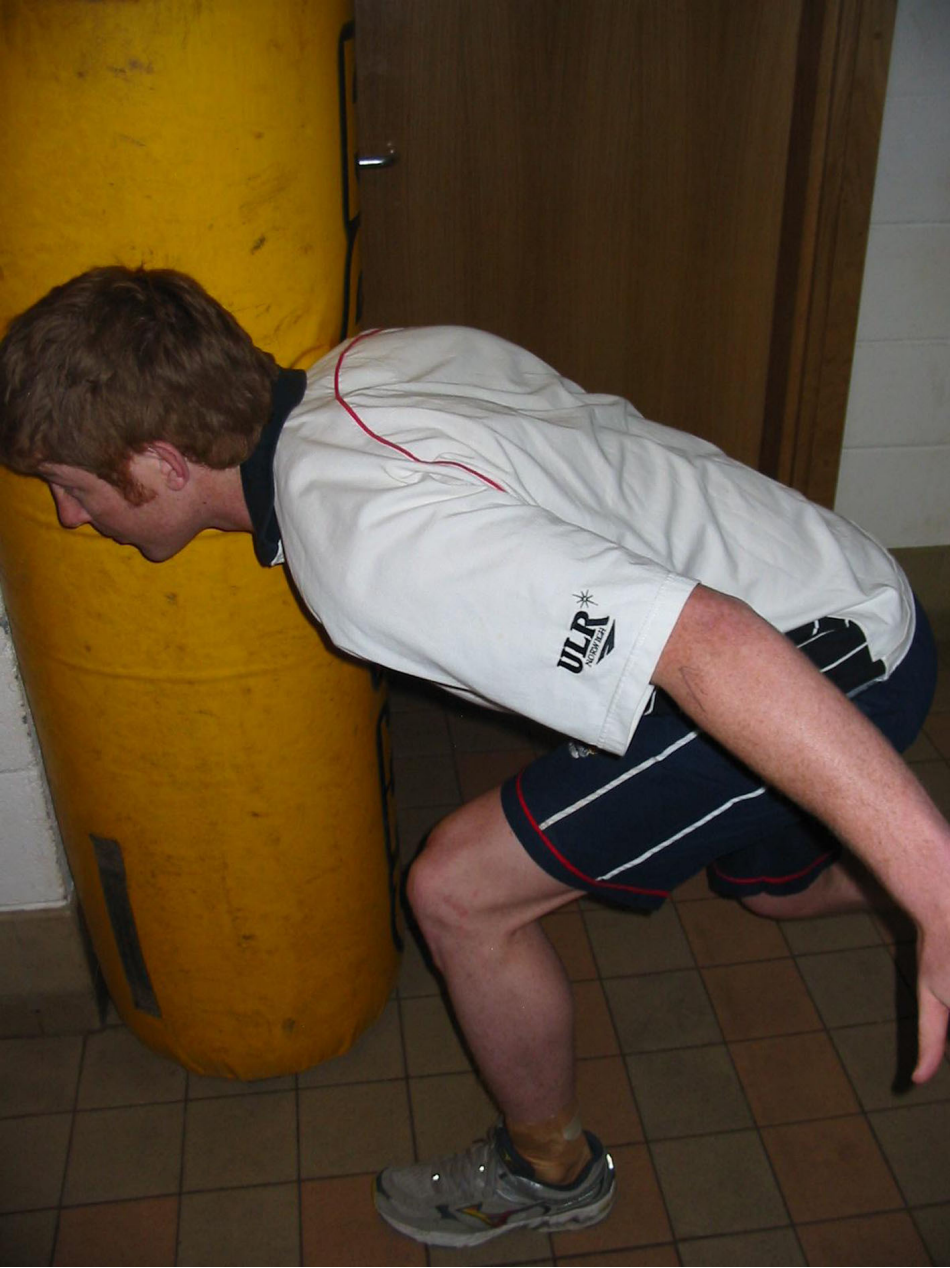
**Shoulder position at contact**.

Each subject aligned the contra-lateral foot to the tackling shoulder 1 step away from the tackle bag, the trunk was flexed to approximately a 90 degree angle between the trunk and thigh, knees flexed to 45 degree and shoulder abducted to about 60 degree (Figure 3). Upon a command from the investigator, the subject prepared on the word "set" and then on the command "hit" (with a 2 second delay between each command, the player pushed forwards through the legs, extending at the hips and knees (but keeping their feet in place) and hit the tackle bag with maximal volitional force, with the chosen shoulder (Figure 4). The EMG data was recorded from the command "hit" until contact was made with the tackle bag. This was repeated 5 times for each shoulder, with a 60 second rest between each repetition.

### Analysis

Data were analyzed using the statistical software package SPSS (version 12). Differences in time of onset between muscles were analyzed with a factorial ANOVA with two factors (side and muscle). The critical alpha level chosen α = 0.05. Paired t-tests were used to evaluate specific differences found (corrected for family-wise inflation of type 1 error with Bonferroni corrections). In order to assess the test-retest reliability of the muscle onset timing, the second and the fifth repetition for each subject for all muscles was compared using intra class correlation coefficient (ICC) to assess both the degree of correspondence and agreement between the tests [46]. Measurement variability was calculated using 95% confidence limits (CI) using the formula [47].

Table 1 shows the test-retest reliability of the muscle onset times.

**Table 1:** Test-retest reliability of the muscle onset times

	Pectoralis Major (Msec)	Biceps Brachii (Msec)	Latissimus Dorsi (Msec)	Serratus Anterior (Msec)	Infraspinatus (Msec)
Mean difference	1.7	1.3	1.3	1.9	2.0
Standard Deviation (SD)	1	1	0.6	1.1	1.1
Standard error of measurement (SEM)	0.33	0.39	0.22	0.35	0.4
Confidence interval (95%)	1.06-2.34	0.87-2.06	0.87-1.73	1.21-2.59	1.22-2.78
ICC_3,k_	0.89*	0.85*	0.87*	0.9*	0.87*

* Statistical Significant (p < 0.01)

95% CI = 1.96 × SEM (54)

SEM = SD × √1-ICC (54)

## Results

Table 2 shows the muscle onset times prior to impact for the injured, uninjured and reference shoulders along with the confidence intervals for these measurements. The larger the time, the longer period the muscle is active prior to impact.

**Table 2:** Onset time prior to impact

	Mean Onset Time Msec (95% CI)		
Muscle	Injured Shoulders	Non-Injured Shoulders	Control Shoulders
**Pectoralis Major**	15.9(9.9-21.9)	23.5(17.5-29.5)	20.7(16.3-25.1)
**Biceps Brachii**	22.7(19.7-25.7)	30(23.2-36.8)	27(23-31)
**Latissimus Dorsi**	25.5(17.1-33.9)	33.6(22.4-44.8)	37.8(35-40.6)
**Serratus Anterior**	38.6(31.6-45.6)	44.6(36.6-52.6)	41.2(38.2-44.2)
**Infraspinatus**	33(22-44)	41.2(30---52.4)	35.4(30.6-40.2)

### Within subject comparison of onset times in the SLAP group

The 2-way factorial ANOVA for within subject comparison indicated a significant group (injured, non-injured) by muscle (Pectoralis Major, Biceps, Latissimus Dorsi, Serratus Anterior, Infraspinatus) interaction (p = 0.01). The main effects of muscle (p = 0.0001) and limb status (p = 0.007) showed significant differences. Paired t-tests were undertaken to evaluate if any specific differences occurred between the individual muscles and injured and non-injured limbs.

Paired t-tests indicated that for the non-injured shoulders, Serratus Anterior was activated prior to all other muscles (p < 0.024), with the exception of Infraspinatus (p = 0.54), which itself had significantly earlier activation than Pectoralis Major (p = 0.024). Comparison between all other muscles for the non injured shoulders showed no significant differences (p > 0.05) in activation time. It should be noted here that the activation timing followed a very similar pattern in the control shoulders, Serratus Anterior was activated prior to all other muscles (p < 0.003), with the exception of Infraspinatus (p = 0.14), which itself had significantly earlier activation than Pectoralis Major (p = 0.0001) and Latissimus Dorsi (p = 0.03). Comparison between all other muscles for the control shoulders showed no significant differences (p > 0.05) in activation time

In the SLAP injured shoulder Serratus anterior was activated significantly earlier than all other muscles (p < 0.03) with the exception of Latissimus Dorsi where no significant difference occurred (p = 0.9). Latissimus Dorsi itself was activated significantly earlier than Biceps (p = 0.033).

The onset of Biceps activity was significantly later, within the SLAP injured shoulder, compared with the contra lateral (un-injured) limb, 22.7 msec versus 30 msec (p = 0.0001). This was the only muscle to show significant timing differences between the SLAP injured and uninjured contra lateral limb.

### Between subject comparison of onset times

The 2-way factorial ANOVA for the between subject comparison indicated a significant group (injured, non-injured, control) by muscle (Pectoralis Major, Biceps, Latissimus Dorsi, Serratus Anterior, Infraspinatus) interaction (p = 0.018). The main effects of muscle (p = 0.0001) and limb status (p = 0.05) showed significant differences.

Paired t-tests were undertaken to evaluate if any specific differences occurred between the individual muscles and injured and non-injured limbs. Paired t-tests (corrected for family-wise inflation of type 1 error with Bonferroni corrections) indicated that biceps activation was significantly delayed in the SLAP shoulder compared to the contra lateral and control shoulders (p < 0.01). Comparison between all other muscles showed no significant differences (p > 0.05) in activation timing. The onset times of all muscles of the non-injured shoulder of the injured players showed no significant difference in activation timing than the muscles of the shoulders of the reference group. The confidence intervals for the control group were quite narrow, which shows consistency of data, and those of the injured and un-injured shoulder show a wide variation.

## Discussion

In the study undertaken it was found that in all shoulders assessed, the onset of Serratus Anterior muscle activity occurred significantly earlier than all other muscles examined, with the exception of Latissimus Dorsi in the injured shoulder and Infraspinatus in the uninjured and control shoulders.

Glousman and co-workers [48], when examining muscle recruitment of elite baseball pitchers, found that throughout the full pitching cycle, athletes with anterior shoulder instability had a reduced activity of their Serratus Anterior compared to normals. The acceleration phase of the pitch can be likened to the tackle position, whereby the humerus internally rotates, and the angular velocity of the glenohumeral joint is increased by the activity of, amongst other muscles, Latissimus Dorsi. During this phase Latissimus Dorsi must contract eccentrically to decelerate horizontal adduction, and resist shoulder distraction and anterior subluxation forces [49]. It has been postulated by Poulliart and Gagey [50] following their cadaveric review of the anatomy of the Latissimus Dorsi, that the muscle, due to the hammock formed by the tendon anterior to the humeral head, may restrain the head when it is subjected to a dislocating force in abduction. Hence we postulate that Latissimus Dorsi compensated for the anterior instability by being recruited earlier to combat the earlier onset of Pectoralis Major, which-due to its attachment in front of the centre of rotation of the glenohumeral joint, would produce anterior shear of the humeral head.

Any delay in the activity of Serratus Anterior could impair scapular control e.g. lateral (upward) rotation and protraction. This would allow the humeral head to translate anteriorly and superiorly [51] when the humerus reached an abducted position at the tackle. Kibler [46] described the mechanism whereby as the humeral head moves on the glenoid, the scapula rotates simultaneously, thereby maintaining the correct relative positions of the scapula and humerus. This positioning is responsible for providing the optimal length-tension relationship of the rotator cuff. A resultant loss of an optimal length-tension relationship within the rotator cuff muscles could detrimentally affect the dynamic stability of the glenohumeral joint.

It has been previously hypothesized that failure to maintain the correct humeral-glenoid alignment could then be responsible for causing a SLAP lesion within the glenohumeral joint [52]. Interestingly, the findings of this study would appear to indicate activation timing of the Serratus Anterior muscle may not be an issue with this particular population of athletes, as there was no significant difference in timing of Serratus Anterior activation occurring between the groups. In addition, this study found Serratus Anterior to be active significantly earlier than the other muscles tested.

These results are in contrast to other research published; Scovazzo and colleagues [53] reported a significant delay in Serratus Anterior activity in front crawl swimmers with shoulder pain, Wadsworth and Bullock-Saxton [54] identified varied EMG activity in Serratus Anterior within injured swimmers compared to asymptomatic swimmers, Glousman and co-workers [48] identified that within elite baseball pitchers with anterior instability, there was reduced Serratus Anterior activity in all phases of throwing when compared to normals, and McMahon and colleagues reported that within the shoulders of athletes with anterior instability there was reduced activity of Serratus Anterior when compared to normals [55]. There are several explanations for this difference. It could be due to the fact that the subjects in this study did not experience pain when carrying out the tackle task, whereas the subjects in these studies complained of pain during their activity. It may also be due to the fact that Serratus Anterior has been reported as being more active when performing movements which simultaneously create upward scapular rotation and protraction [56]. The starting position of the subjects in this study may also have implications for the onset of Serratus Anterior as the shoulder was preset prior to the movement into the tackle.

This absence of any difference in timing may indicate that Serratus Anterior dysfunction may not have a role in the injury mechanism of SLAP lesions associated with a tackle activity, although additional work would be needed to truly confirm this, as this study has a relatively small sample size.

Many researchers [57-59] have demonstrated the preparatory hamstring muscle activity within the knees of ACL deficient patients. This produces muscle stiffness which then increases muscle spindle sensitivity and reduces EMD. Solomon *et al*. [60] have demonstrated the existence of a spinal reflex between the shoulder capsule and the shoulder muscles within the feline model, which was demonstrated within the human shoulder by Jerosch *et al*. [61]. Although they postulated that this reflex was too slow to provide joint stabilization, previous research has shown that pre-activation of muscles (in this case, around the shoulder joint) may provide a rapid compensation in response to external forces, and thus provide joint stability [62]. David *et al*. [63] identified feed forward mechanisms within the rotator cuff occurring prior to both internal or external rotation of the humerus, and Fleisig *et al*. [64] reported that when the humerus is in internal rotation in abduction, the long head of biceps moves anteriorly, providing a compressive force and increasing the anterior stability of the joint as in this position the long head of biceps affords a posteriorly directed force. They also demonstrated that in shoulders with SLAP lesions there was a greater muscle activity from biceps which could be responsible for producing increased glenohumeral joint stability. This increased activity could itself, over time, result in the formation of a superior labral tear.

The early activation of Infraspinatus muscle, in the uninjured and control shoulders, is in line with previous research of Saha [65] who utilized EMG to demonstrate that both Infraspinatus and Subscapularis contracted during mid range elevation to produce glenohumeral stability, and the work of Oveson and Nielson [66] who stated that Infraspinatus helped prevent posterior translation of the humeral head due to its posterior location, aiding posterior joint stability especially in the mid range of 45-75 degrees of abduction. This may explain the findings within the control and uninjured shoulders. This early activity is to be expected as this contraction of a member of the rotator cuff pre-empting movement with stabilizer the humeral head in the glenoid cavity, as during movement at the shoulder the rotator cuff muscles function in a coordinated manner to maintain the humeral head within the glenoid fossa [67]. The control of muscle timing has been termed *temporal recruitment *[62]. This significantly earlier activation of Infraspinatus was absent in the SLAP injured shoulder and may indicate a failure of the local control system so possibly leading to increased stress on the shoulder support structures. Although there was no pain associated with the tackle demand in this study, Hess and co-workers [68] found a significant delay in the onset of Subscapularis when subjecting their pain complaining subjects to rapid external rotation demands, and postulated is was due to a lack of feed forward from the Subscapularis which, in their study, activated 50 milliseconds prior to movement at the shoulder. A similar explanation could be postulated for the delay in activation of Infraspinatus in our study.

### Limitations of paper

Whilst this study has provided information on the recruitment patterns of some of the muscles around the shoulder during a tackle task, only a small sample size was recruited and this sample size could not be matched for position or body mass index.

While the assessment utilized easily available muscles for sEMG, other muscles could have been utilized, with possibly greater accuracy. Also the study was carried out in an artificial environment with all movement in one plane, and does not necessarily demonstrate what happens on the field of play where there are force vectors from many directions, and increased momentum within the tackle.

This study does not provide information as to whether this recruitment pattern occurs as a result of injury to the labrum or whether it is a causative factor in the development of type II SLAP lesions. It could be that the alteration in muscle onset timing is a mechanism to avoid pain during tackling.

### Clinical implications

The over activity of Latissimus Dorsi needs discouraging, as this compensatory mechanism may produce abnormal muscle patterning which could lead to further, possibly inferior, instability around the glenohumeral joint.

If the delay in onset of Infraspinatus recruitment continues this could also lead to increased ligamentous strain, especially during external rotation, resulting in a possible lack of anterior stability during humeral abduction. Any form of muscle imbalance within the rotator cuff could lead to increased instability [69].

In comparison to other studies which have identified a delay in activation of Serratus Anterior, in painful unstable shoulders, this study indicates that facilitation of the Serratus Anterior may not be necessary in the case of rugby players with type II SLAP lesions, as there is no significant delay reported. Moreover it may be pertinent to direct rehabilitation to facilitate the onset of Biceps and Infraspinatus and inhibit the early onset of Latissimus Dorsi.

## Conclusions

This study shows that in shoulders with a SLAP lesion there is a trend towards delay in activation time of Biceps and other muscles with the exception of an associated earlier onset of activation of Serratus anterior, possibly due to a coping strategy to protect glenohumeral stability and thoraco-scapular stability. This trend was not statistically significant in all cases.

## Competing interests

The authors declare that they have no competing interests.

## Authors' contributions

IH and LH were fully involved in the design, data acquisition and analysis for the paper. All authors (IH, LH, CR) were fully involved in the conception and drafting of the paper related to the study. All authors read and approved the final manuscript.
